# Ephedrae Herba-Associated Adverse Events: A Disproportionality Analysis Integrated with Network Pharmacology

**DOI:** 10.3390/ph19091340

**Published:** 2026-08-24

**Authors:** Musun Park, Hyeun-Kyoo Shin, Yujin Choi

**Affiliations:** 1 KM Data Division, Korea Institute of Oriental Medicine, Daejeon 34054, Republic of Korea; bmusun@kiom.re.kr; 2 KM Science Research Division, Korea Institute of Oriental Medicine, Daejeon 34054, Republic of Korea; hkshin@kiom.re.kr; 3 NeuroGlymph Imaging and Modulation Center, Korea Institute of Oriental Medicine, Daejeon 34054, Republic of Korea

**Keywords:** Ephedrae Herba, pharmacovigilance, adverse events, disproportionality analysis, network pharmacology, KAERS DB

## Abstract

**Background/Objectives**: Ephedrae Herba (EH) is widely used in traditional East Asian medicine, but safety concerns regarding its adverse events remain. This study aimed to investigate EH-associated adverse events using clinical pharmacovigilance data and to perform exploratory in silico analyses to propose potential molecular mechanisms underlying these adverse events. **Methods**: A disproportionality analysis was performed using individual case safety reports from the Korea Adverse Event Reporting System database. EH-containing products were compared with other herbal medicine products using reporting odds ratios (RORs), proportional reporting ratios, and information components. Network pharmacology identified adverse event-related genes and pathways, and protein–protein interaction networks were constructed. Molecular docking predicted direct adverse event-associated targets of ephedrine and compared mechanisms with control compounds (aconitine and spinosin). **Results**: Four adverse-event signals were detected in the primary analysis: sleep disorder, dry mouth, insomnia, and palpitations. Sensitivity analysis identified four signals, with three (dry mouth, insomnia, and palpitations) consistent across both analyses; constipation emerged only in the sensitivity analysis. **Conclusions:** Adverse event-associated network analysis predicted key pathways: Neuroactive ligand–receptor interaction, Pathways of neurodegeneration, and Dopaminergic synapse. Molecular docking predicted that ephedrine may act on downstream signaling mechanisms shared by neurotransmitter systems, including the dopaminergic system, distinct from control compounds.

## 1. Introduction

Ephedrae Herba (EH), the dried aerial stem of *Ephedra* species, has been used in traditional East Asian medicine for over 2000 years to treat respiratory conditions, fever, and related ailments [1,2]. The primary bioactive constituent, ephedrine, acts as a sympathomimetic agent through both direct stimulation of α- and β-adrenergic receptors and indirect enhancement of norepinephrine release [3,4].

Despite EH’s long history of traditional use, safety concerns have emerged, particularly from reports of adverse cardiovascular and central nervous system events associated with ephedrine-containing dietary supplements [5,6]. These reports led the U.S. Food and Drug Administration to ban the sale of dietary supplements containing ephedrine alkaloids in 2004 [7]. However, EH-containing herbal formulations continue to be used in Korea and other East Asian countries within traditional medicine systems, in which they are typically combined with other herbs for various therapeutic purposes, including respiratory conditions and weight loss [2,8,9,10,11].

While spontaneous reporting systems serve as the cornerstone for postmarketing pharmacovigilance, most herbal medicine safety studies have focused primarily on signal detection without investigating the underlying molecular mechanisms [12,13]. Understanding the mechanistic basis of adverse events is essential for predicting the safety profiles of herbal combinations and optimizing clinical use. To address this issue, network pharmacology (NP) offers a promising method for bridging clinical safety signals with molecular-level mechanisms [14,15,16]. Based on network biology, NP reflects the functional characteristics of interaction-based protein modules, allowing the identification of candidate biological processes associated with adverse events [17]. In addition, integrating NP with in silico approaches such as molecular docking enables prediction of potential molecular interaction sites and associated signaling clusters underlying EH-related adverse events [18]. Consequently, NP-based analyses that include molecular docking for exploring biological mechanisms provide a valuable framework for clinical application and the systematic exploration of adverse events.

This study aims to identify adverse event signals associated with EH-containing products using disproportionality analysis of the Korea Adverse Event Reporting System (KAERS) database and to explore the potential molecular mechanisms underlying EH-associated adverse events by integrating NP and molecular docking analyses as a hypothesis-generating approach.

## 2. Results

### 2.1. Study Population

#### 2.1.1. Selection of Individual Case Safety Reports

From the KAERS database, 1054 individual case safety reports (ICSRs) related to 84 herbal medicine products reported between 2012 and 2021 were initially identified. Of the 84 products, 53 had one or more adverse event reports. Of the 1054 reports, 30 duplicate reports were excluded (retaining only the most recent follow-up report for each case), and one report with a missing adverse event name was excluded, resulting in 1023 ICSRs for analysis. Seven EH-containing products were identified from the 53 products with ICSRs (Table A1). A total of 379 reports (37.0%) were associated with these seven EH-containing products. Of these reports, EH was classified as a suspected drug in 335 (88.4%) and as a concomitant medication in 44 (11.6%).

#### 2.1.2. Baseline Characteristics of the Reports

**Figure 1:**
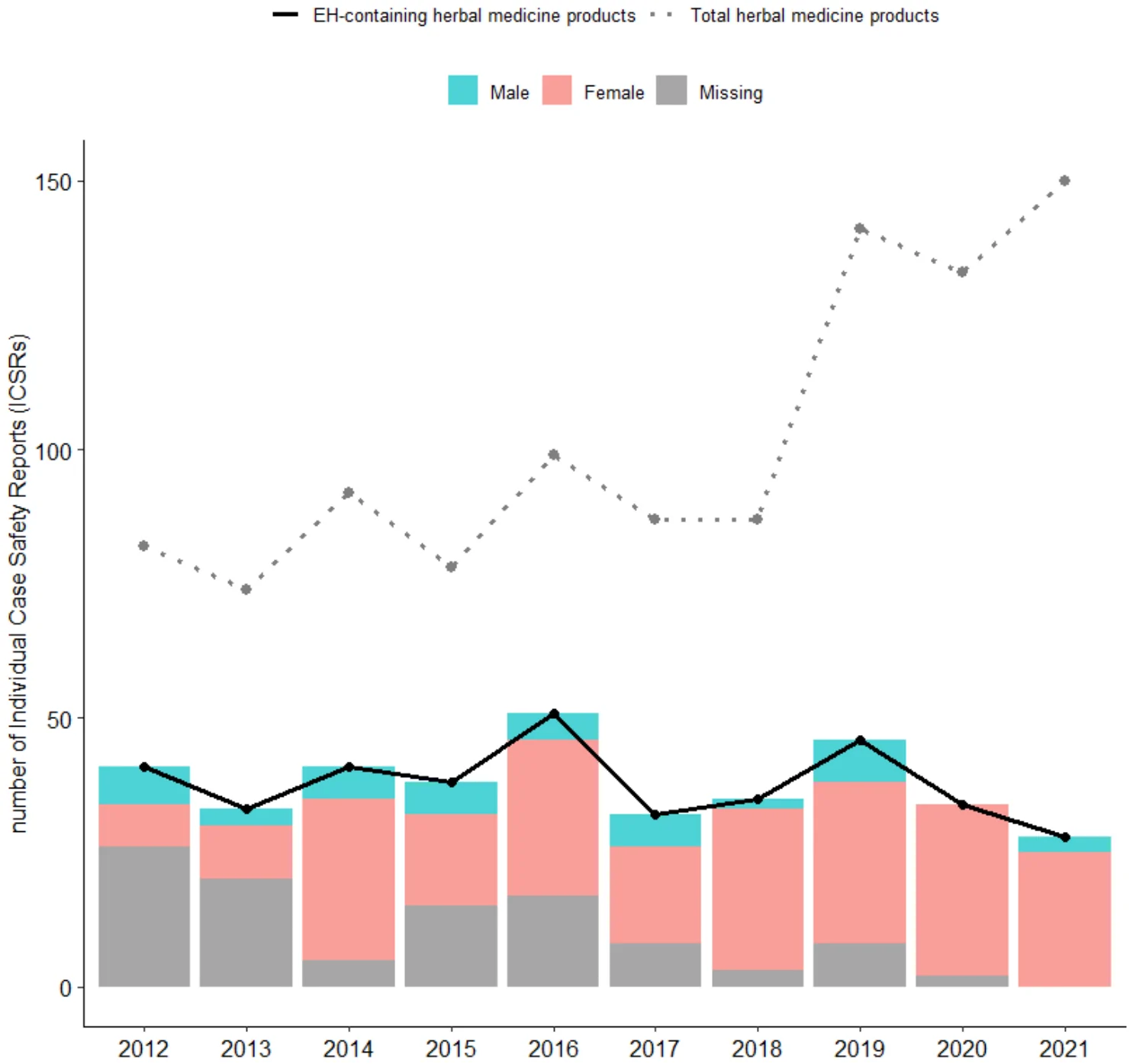
Annual number of individual case safety reports. EH-containing herbal medicine products (bar plot and solid line plot) and total herbal medicine products (dotted line plot). EH, Ephedrae Herba.

The baseline characteristics of the 379 EH-related ICSRs are presented in Table 1, with comparisons with reports for other herbal medicine products (*n* = 644) as a reference group. Annual reporting trends are shown in Figure 1. For EH-related reports, the original reporters were predominantly pharmacists or herbal medicine pharmacists (60.7% vs. 37.0% for other products), followed by consumers/nonmedical professionals (19.0%) and doctors/dentists/Korean medicine doctors (10.3%). Regional pharmacovigilance centers were the reporters in 50.9% of cases, with pharmaceutical companies accounting for 35.1%. The majority of reports were spontaneous (92.9%). Patient demographics showed that females were more frequently affected than males (60.4% vs. 12.1%); sex was missing in 27.4% of EH-related reports, compared with 8.2% of comparator reports. The majority of patients were aged 19–64 years (61.2%), with a notably lower proportion of patients aged 65 years or older compared with other herbal products (7.4% vs. 19.3%).

### 2.2. Disproportionality Analysis

#### 2.2.1. Frequency of Adverse Events

A total of 559 adverse events were reported in the 379 EH-related ICSRs. The distribution of adverse events by System Organ Class (SOC) is presented in Table 2. Gastrointestinal disorders were the most frequently reported (180 events, 32.2%), with common symptoms including abdominal discomfort, diarrhea, abdominal pain, and constipation. Nervous system disorders were the second most common (75 events, 13.4%), primarily consisting of headache and dizziness. Skin and subcutaneous tissue disorders (71 events, 12.7%) and psychiatric disorders (67 events, 12.0%) were also frequently reported, with the latter predominantly comprising insomnia and sleep disorders. The majority of adverse events were classified as nonserious (543 events, 97.1%), while 16 events (2.9%) were classified as serious. Detailed characteristics of these serious adverse events, including seriousness criteria, clinical outcomes, and causality assessments, are presented in Table A2.

The 16 most frequently reported adverse events at the Preferred Terms (PT) level are shown in Table 3. Insomnia was the most common individual adverse event (47 events, 8.4%), followed by abdominal discomfort (36 events, 6.4%), headache (28 events, 5.0%), palpitations (25 events, 4.5%), and diarrhea (24 events, 4.3%).

#### 2.2.2. Signal Detection Results

**Figure 2:**
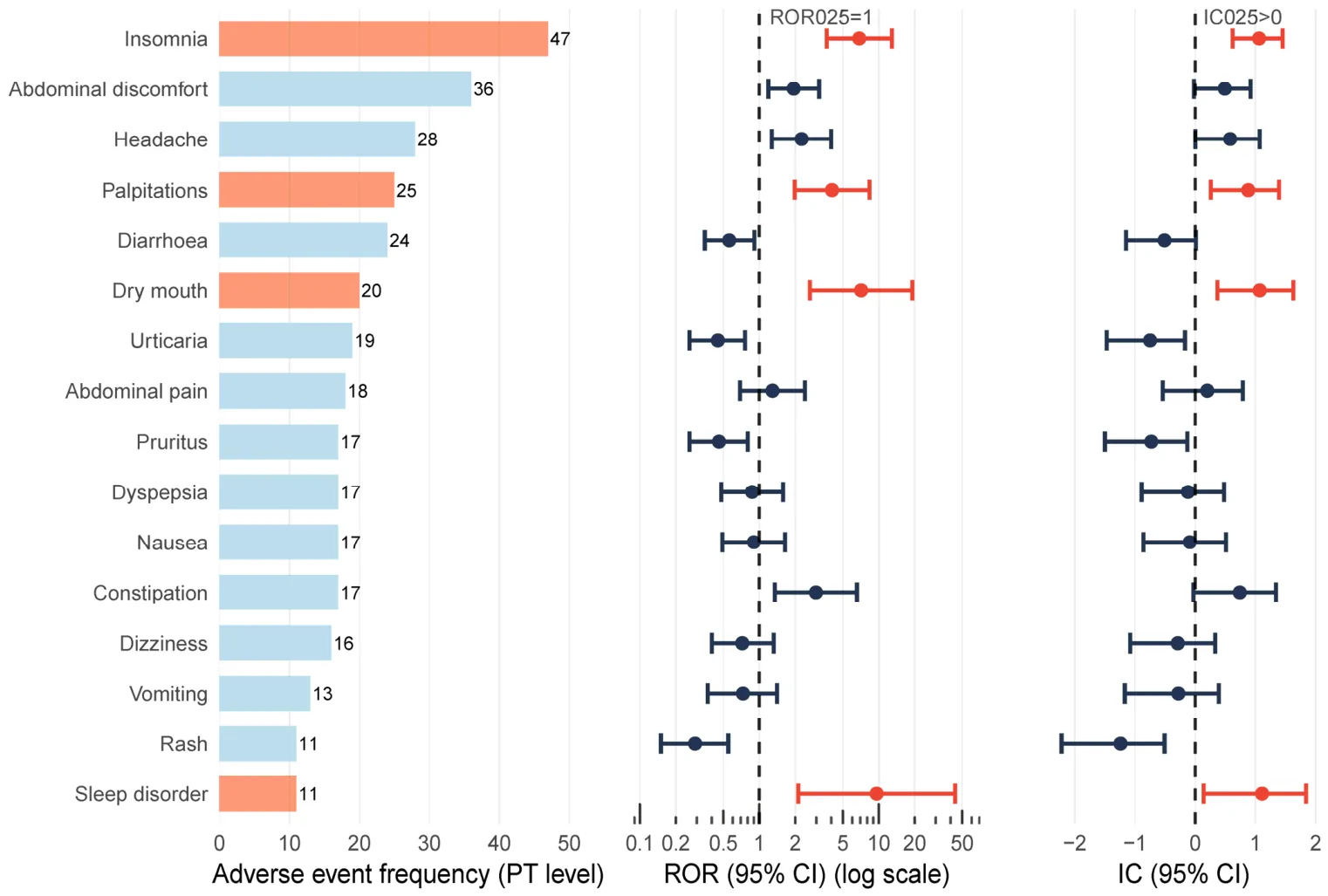
Detected signals of adverse events associated with EH-containing herbal medicine products. Signal detection criteria were defined as a lower limit of the ROR 95% CI ≥ 1 and a lower limit of the IC 95% CI > 0. Red indicates adverse events meeting both signal detection criteria; blue indicates those that did not. EH, Ephedrae Herba.

Disproportionality analysis was performed for the 16 most frequently reported adverse events to identify signals of disproportionate reporting for EH-containing products compared with other herbal medicine products. The results, including the proportional reporting ratios (PRR), reporting odds ratios (ROR), and information components (IC) values with their 95% CIs, are presented in Figure 2 and Table A3. Four adverse events met the predefined signal detection criteria (a lower limit of ROR 95% CI ≥ 1 and a lower limit of IC 95% CI > 0): sleep disorder (ROR 9.60, 95% CI 2.12–43.52), dry mouth (ROR 7.12, 95% CI 2.65–19.13), insomnia (ROR 6.87, 95% CI 3.67–12.88), and palpitations (ROR 4.06, 95% CI 1.98–8.36). Of these signals, sleep disorder showed the strongest association, followed by dry mouth and insomnia. Headache (ROR 2.26, 95% CI 1.27–4.00) showed a comparable point estimate but narrowly failed to meet the IC criterion (IC 95% CI: 0.58, −0.0008 to 1.07), and was therefore not classified as a signal.

#### 2.2.3. Sensitivity Analysis

A sensitivity analysis restricted to suspected drug reports (*n* = 335) identified four signals: dry mouth (ROR 11.99, 95% CI 2.71–53.11), insomnia (ROR 8.18, 95% CI 3.92–17.09), palpitations (ROR 4.87, 95% CI 2.24–10.56), and constipation (ROR 3.91, 95% CI 1.59–9.59) (Table A4). Three signals (dry mouth, insomnia, and palpitations) were consistent across both analyses. Constipation emerged as a signal in the sensitivity analysis after borderline evidence was shown in the primary analysis. Sleep disorder did not meet the IC criterion in the sensitivity analysis.

### 2.3. Biological Protein–Protein Interaction (PPI) Network Analysis of EH-Associated Adverse Events

#### 2.3.1. Results of Collection and Analysis of Biological Entities Related to Adverse Events

**Figure 3:**
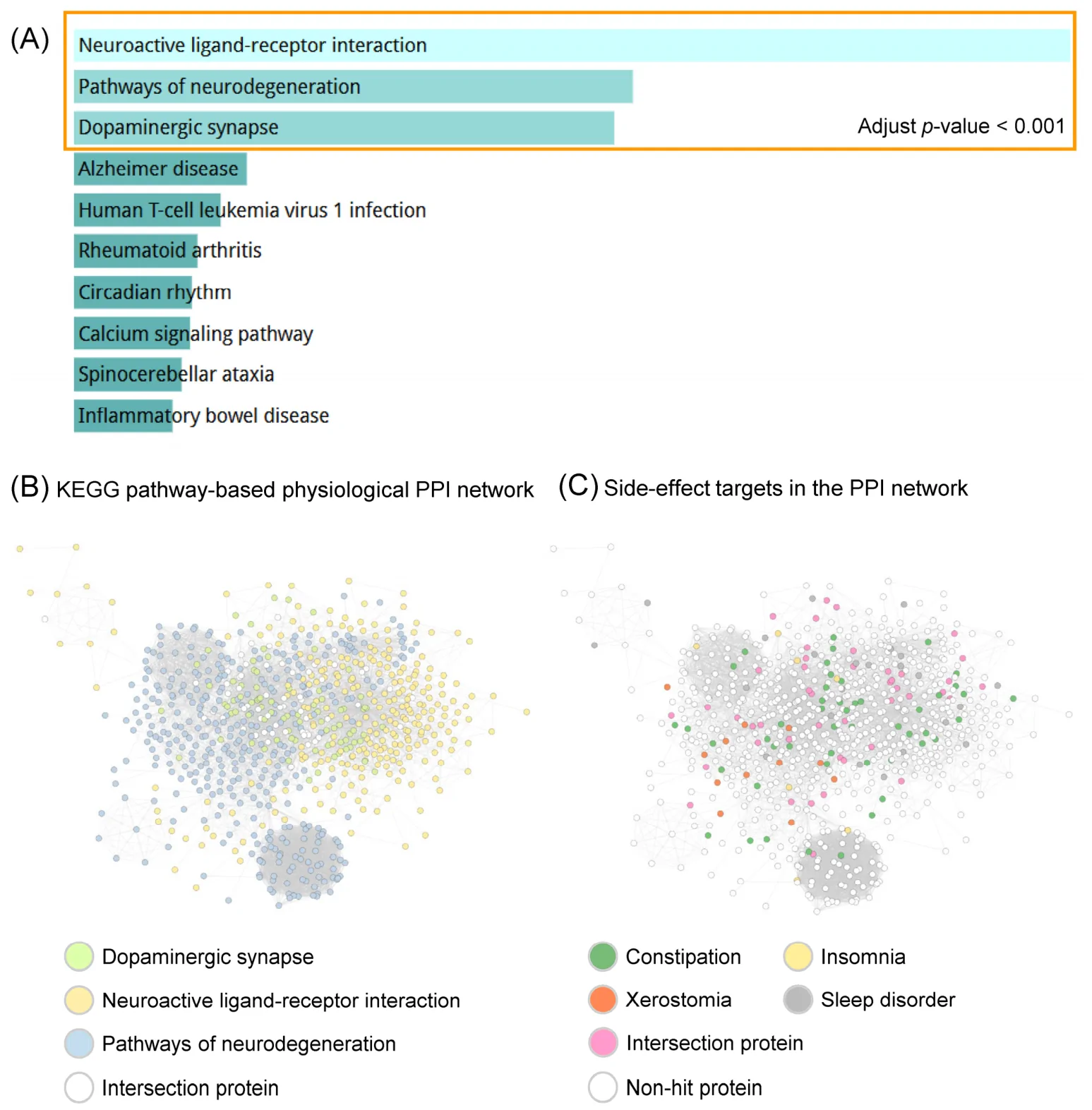
Analysis results using genes related to the adverse effects of EH. (**A**) ORA was performed using proteins that co-occurred in two or more EH-associated adverse effects identified in the previous analysis. The bar graph represents *p*-values, with longer and lighter bars indicating lower *p*-values. The orange box highlights the top three Kyoto Encyclopedia of Genes and Genomes (KEGG) pathways with adjusted *p*-values < 0.001 among the adverse effect-related pathways. (**B**) A pathway-based physiological PPI network constructed using proteins included in the top three KEGG pathways related to adverse effects. (**C**) Color mapping of proteins related to side effect targets on the KEGG pathway-based physiological PPI network. ORA, overrepresentation analysis; PPI, protein–protein interaction; EH, Ephedrae Herba; KEGG, Kyoto Encyclopedia of Genes and Genomes.

Based on the DisGeNET database, 111 genes were identified as insomnia-related, 58 as dry mouth-related, 472 as constipation-related, and 365 as sleep disorder-related (Dataset S1). Although “palpitations” was identified as a significant adverse event signal in the pharmacovigilance analysis, no associated genes were available in the DisGeNET database. Consequently, it could not be included in the downstream network pharmacology analysis. This reflects the coverage of curated gene–adverse event associations available in the publicly available full release of the DisGeNET database used in this study. Of the identified genes, 150 were found to be co-occurring side effect-related genes that were included in two or more side effect groups (Dataset S2). Over-representation analysis (ORA) showed that the enriched pathways were mainly related to the central nervous system (CNS). Among the significantly enriched pathways (adjusted *p*-value < 0.001), Neuroactive ligand–receptor interaction, Pathways of neurodegeneration, and Dopaminergic synapse were identified as significant pathways (Figure 3A, Table A5).

#### 2.3.2. Results of Construction and Analysis of the Adverse Events-Related Biological PPI Network

**Figure A1:**
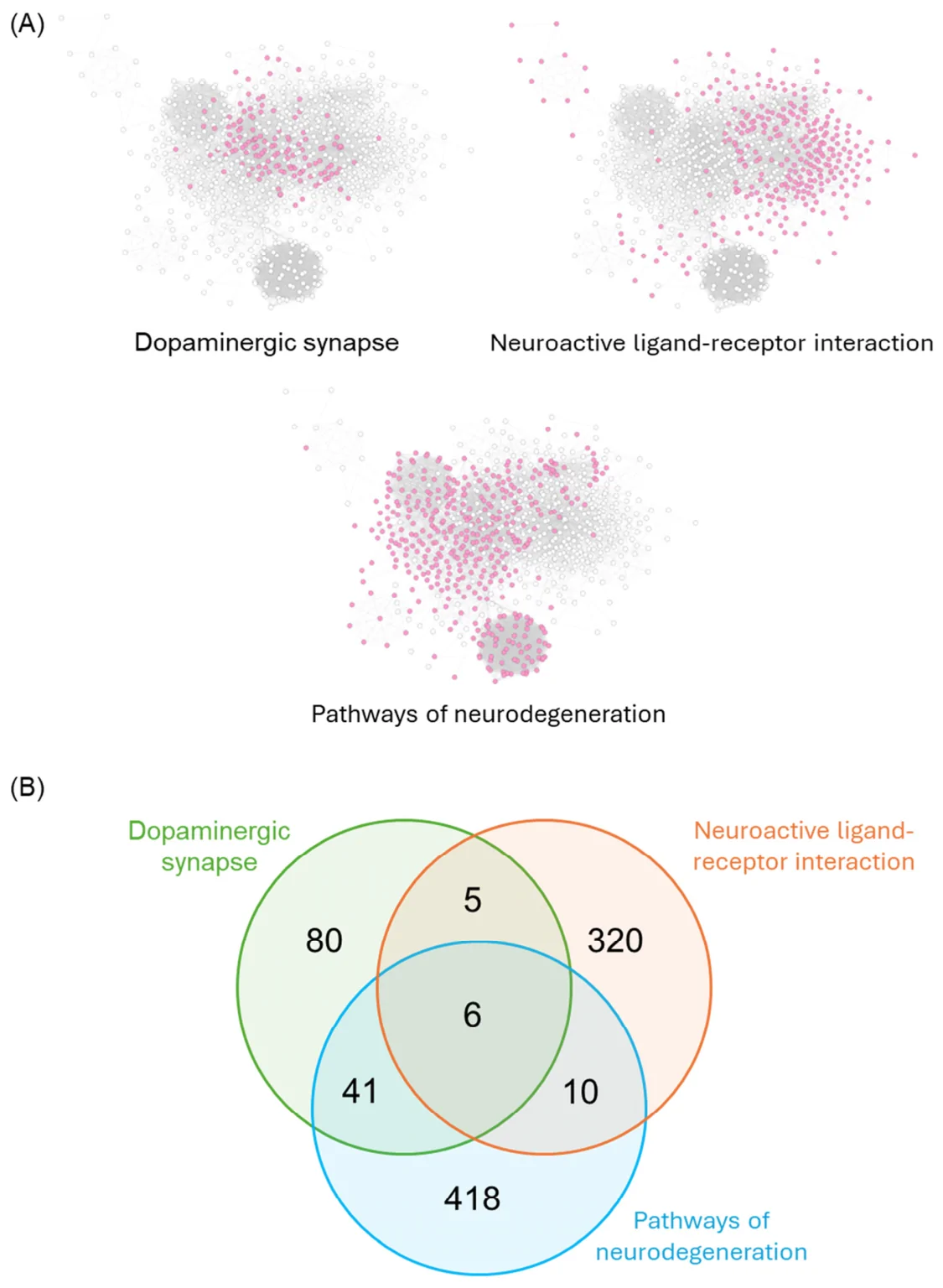
PPI network representing adverse events-related pathways. (**A**) A pathway-based physiological PPI network was constructed using proteins from the top three KEGG pathways related to side effects. Each pathway was color-mapped based on its corresponding gene set. Genes belonging to each pathway were highlighted in pink, while the remaining nodes were shown in gray. (**B**) Venn diagram showing the overlap among the three predicted side effect-related KEGG pathways. PPI, protein–protein interaction; KEGG, Kyoto Encyclopedia of Genes and Genomes.

Construction of the adverse events-related biological PPI network resulted in 762 protein nodes and 6228 edges (Figure 3B, Datasets S3 and S4). Of these nodes, 131 were in the Dopaminergic synapse, 288 in the Neuroactive ligand–receptor interaction, and 410 in Pathways of neurodegeneration (Figure A1A, Dataset S4). In the sensitivity analysis, the average values of degree centrality, betweenness centrality, and closeness centrality were calculated for each group at each confidence threshold. The Dopaminergic synapse group showed the highest values across all three metrics except degree centrality at the highest confidence threshold. In contrast, the Neuroactive ligand–receptor interaction group showed the lowest centrality values across all confidence thresholds (Table A6). A total of 61 proteins overlapped between two or more pathways, whereas 701 proteins were uniquely included in a single pathway, suggesting that the three pathways have distinct and independent roles within the network (Figure A1B).

**Figure A2:**
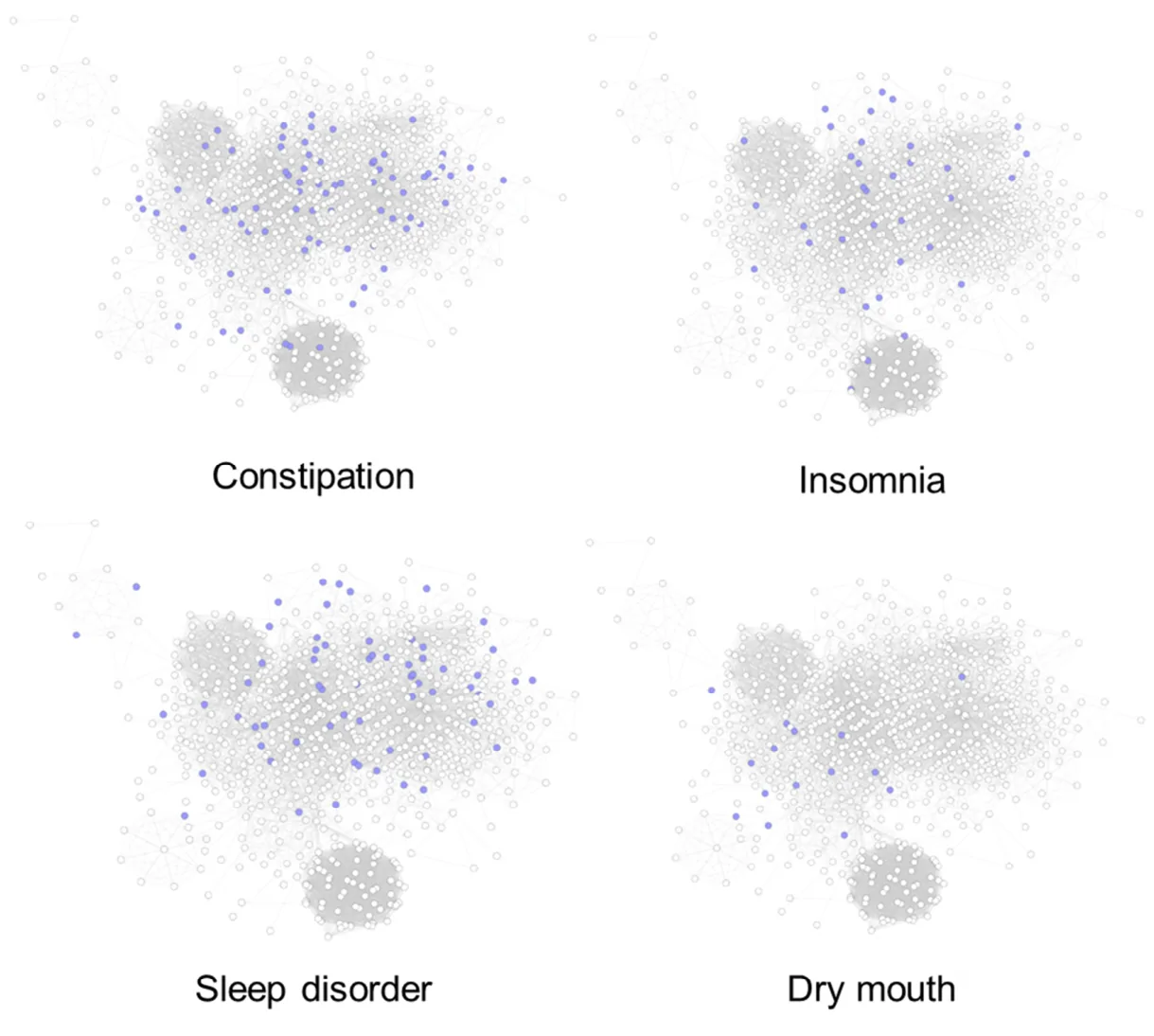
PPI network representing adverse events-related genes. Proteins included in the four adverse events-related gene sets were color-mapped onto the KEGG pathway-based physiological PPI network. PPI, protein–protein interaction; KEGG, Kyoto Encyclopedia of Genes and Genomes.

In the adverse events-related highest confidence biological PPI network, the numbers of proteins related to each side effect were as follows: 76 for constipation, 35 for insomnia, 64 for sleep disorder, and 15 for dry mouth (Dataset S4). Analysis of the distribution of side effect-related proteins within the three pathways showed that they were relatively evenly distributed among all pathways (Figure A2, Table A7). In particular, all 15 proteins associated with dry mouth were found to be related to the Pathways of neurodegeneration.

### 2.4. Results of Exploratory Docking Screening and Network Analyses Using the Major Compound of EH

#### Results of Docking and Network-Based Prediction of Adverse Events-Related Targets

**Figure 4:**
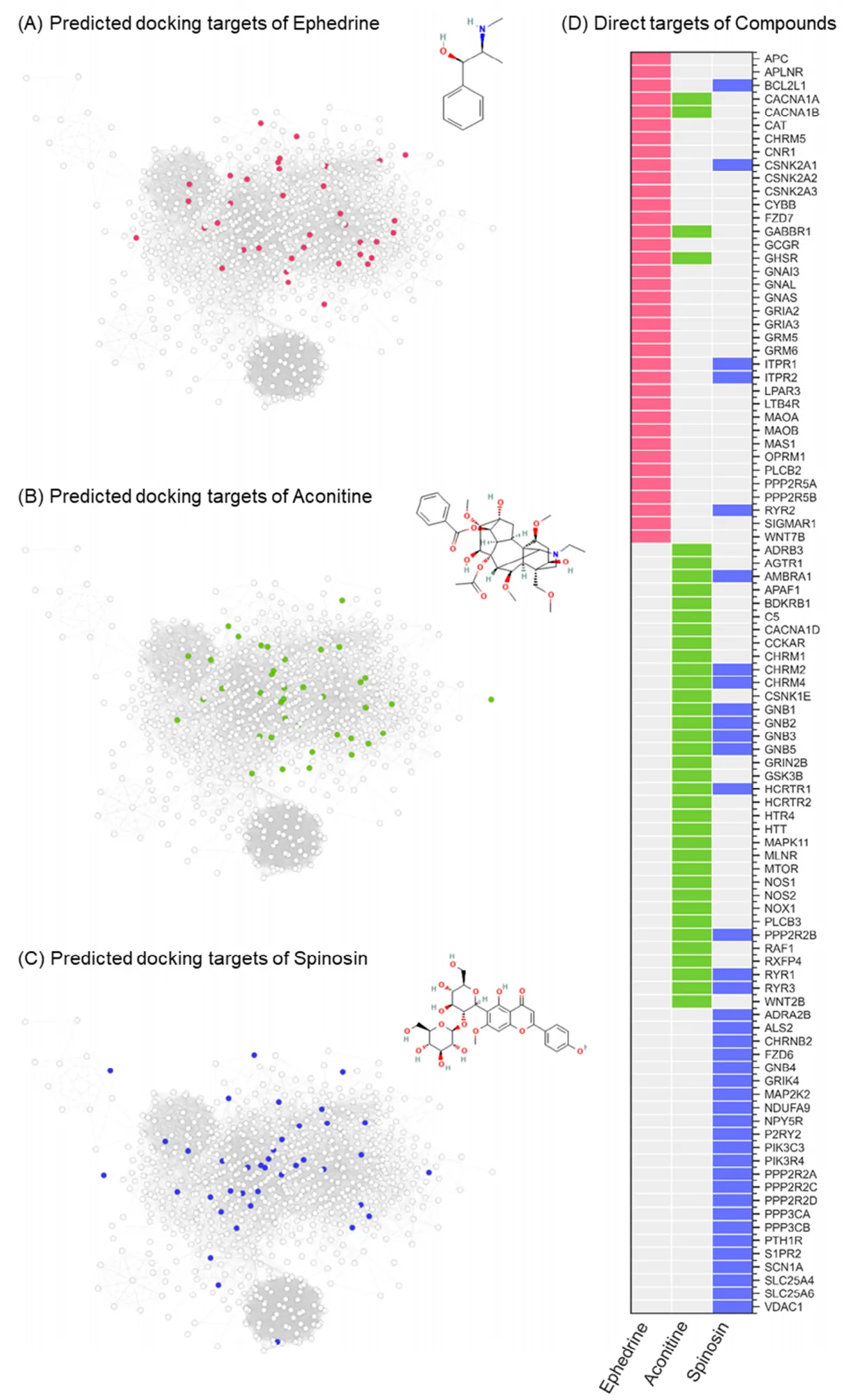
Analysis results of the primary direct targets of ephedrine, aconitine, and spinosin. (**A**–**C**) The top 5% of proteins (empirical *p*-value < 0.05) predicted to have the highest potential for interaction in docking analyses were selected for each compound (ephedrine, aconitine, and spinosin). The selected proteins constituting the KEGG pathway-based physiological PPI network were color-mapped. (**D**) Heatmap visualizing the top 5% of proteins predicted to have high interaction potential with the three compounds. KEGG, Kyoto Encyclopedia of Genes and Genomes; PPI, protein–protein interaction.

**Figure A3:**
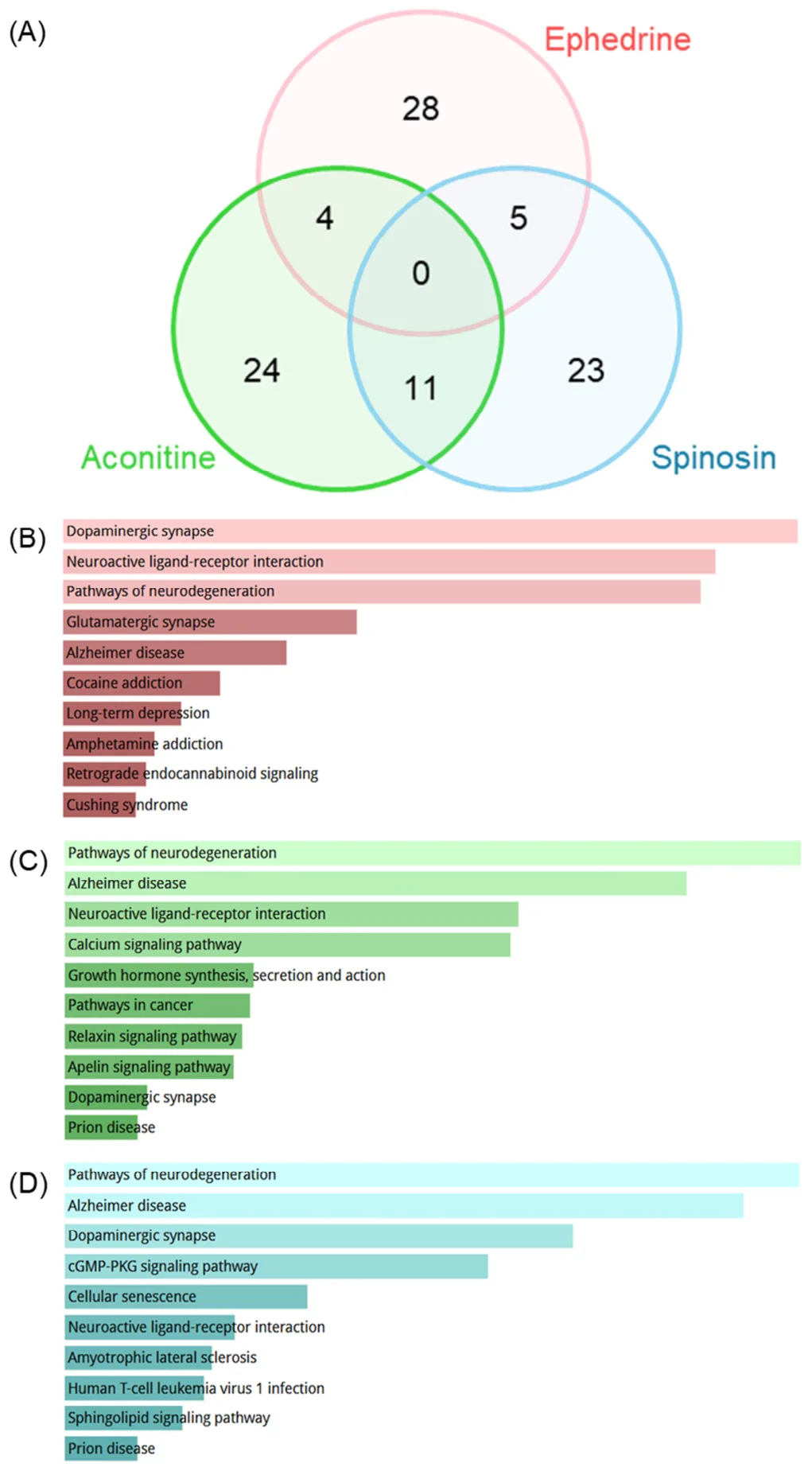
Results of the exploratory docking analysis using the three compounds. (**A**) Venn diagram illustrating the overlapping proteins among the adverse events-related proteins predicted from exploratory docking analysis of the three compounds. (**B**) The ORA results using the 28 proteins predicted to interact exclusively with ephedrine are presented. (**C**) The ORA results using the 24 proteins predicted to interact exclusively with aconitine are presented. (**D**) The ORA results using the 23 proteins predicted to interact exclusively with spinosin are presented. ORA, Over-representation analysis.

Using the 762 proteins included in the three pathways, an exploratory docking analysis was performed to predict the direct molecular targets of the representative compounds from the three herbs. We used the Score-Integrated Ligand Efficiency (SILE) binding affinity scores derived from the exploratory docking analysis not as absolute measures of interaction strength, but rather as relative indicators to predict potential target proteins. Therefore, in this study, the quantitative values of the binding affinity scores were not emphasized, while the scores normalized using SILE were provided in the Supplementary Dataset (Dataset S5). Among the proteins showing significant interaction potential (empirical *p* < 0.05), those included in the PPI network were predicted as follows: 37 for ephedrine, 39 for aconitine, and 39 for spinosin (Figure 4, Dataset S5). Centrality analysis using sensitivity analyses showed that ephedrine generally exhibited lower centrality values than aconitine and spinosin. In addition, non-interaction nodes showed lower centrality values across all metrics except degree centrality at the highest confidence threshold, suggesting that proteins predicted to interact with each compound had a relatively higher potential to act as hubs within the network (Figure 4, Table A8). Among the predicted targets of each compound, 20 proteins overlapped between two or more compounds. More than 50% of the predicted proteins for each compound did not overlap with those of the others, indicating that each compound may act through relatively independent mechanisms (Figure 4D and Figure A3A).

**Figure 5:**
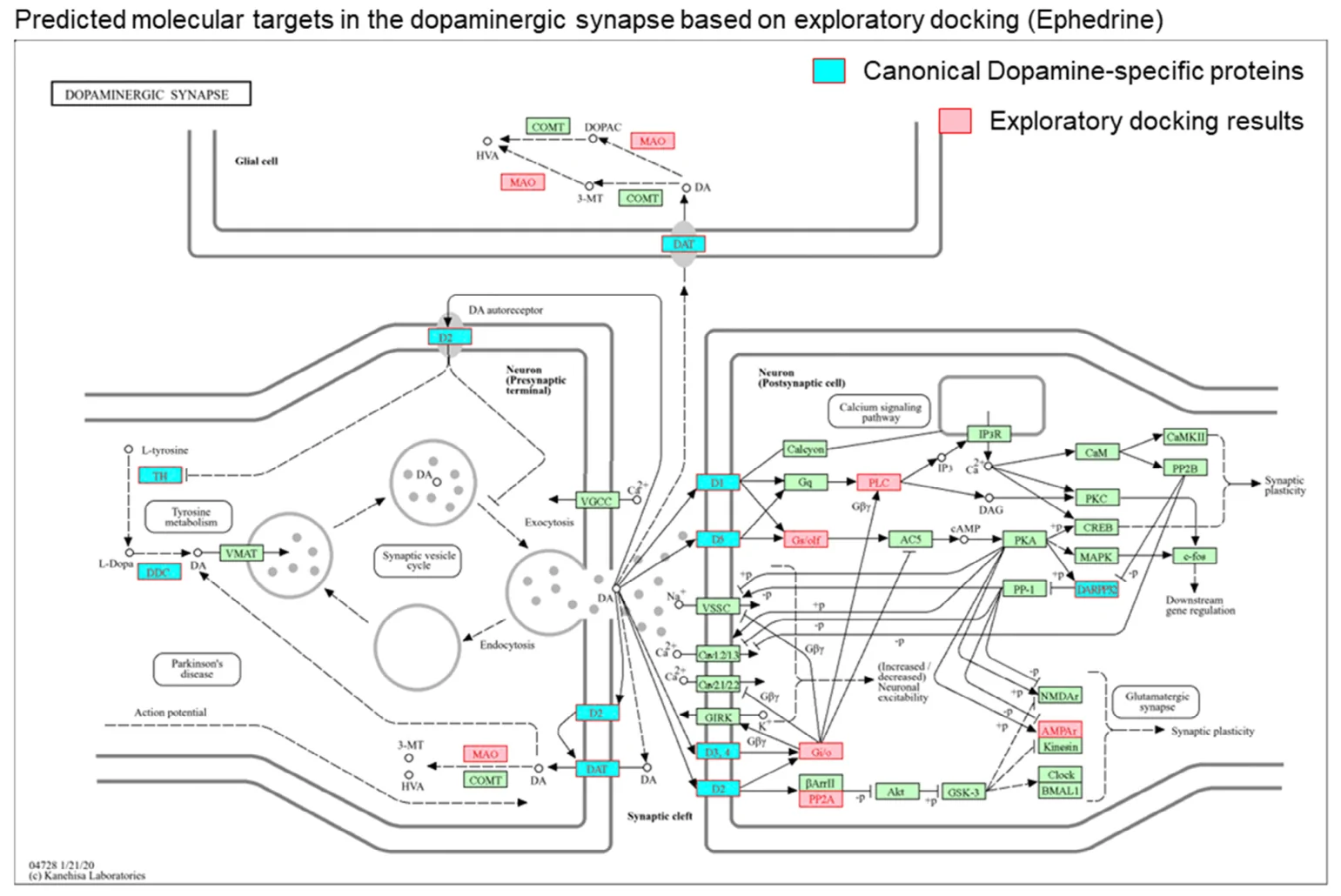
Predicted molecular targets of ephedrine in the dopaminergic synapse based on exploratory docking. Proteins predicted to be specifically affected by ephedrine within the dopaminergic synapse pathway are presented. Red boxes indicate the proteins predicted to interact with ephedrine, whereas blue boxes indicate dopamine−specific proteins. Using KEGG Mapper, the locations of the target proteins predicted to be directly affected by ephedrine within the dopaminergic synapse pathway were visualized. KEGG, Kyoto Encyclopedia of Genes and Genomes.

**Figure A4:**
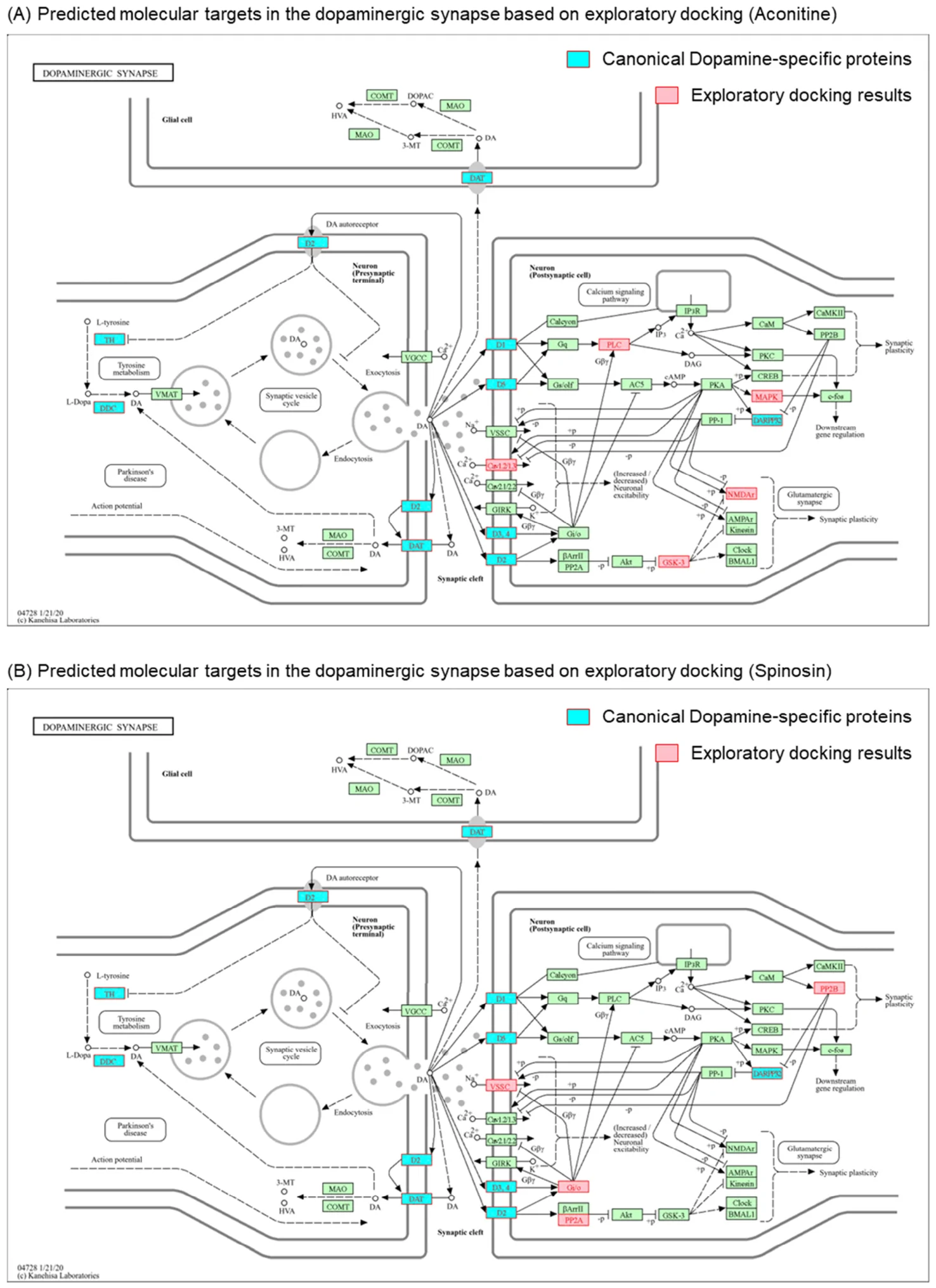
Predicted molecular targets of aconitine and spinosin in the dopaminergic synapse based on exploratory docking. Proteins predicted to be specifically affected by aconitine and spinosin within the dopaminergic synapse pathway are presented. (**A**) presents the results for aconitine, and (**B**) presents the results for spinosin. Red boxes indicate the proteins predicted to interact with each compound, whereas blue boxes indicate dopamine−specific proteins. Using KEGG Mapper, the locations of the target proteins predicted to be directly affected by each compound within the dopaminergic synapse pathway were visualized. KEGG, Kyoto Encyclopedia of Genes and Genomes.

ORA using proteins predicted to interact exclusively with each compound showed that aconitine and spinosin were most likely to act on the Pathways of neurodegeneration, whereas ephedrine, unlike the other two compounds, was most likely to act on the Dopaminergic synapse (Figure A3B–D). The proteins predicted through the in silico analysis were associated with the pathophysiology of the disease. However, not all proteins directly induce the disease itself. Therefore, to further compare the mechanisms that are more likely to induce perturbation of the protein cascade, the direct molecular targets of each compound were mapped onto the Dopaminergic synapse pathway, which was predicted to be specifically affected by ephedrine. (Figure 5). In the dopaminergic synapse, dopamine binds to canonical dopamine-specific receptors and subsequently transmits signals through downstream signaling pathways. The analysis of ephedrine predicted potential interactions with proteins such as GNAL, GNAS, MAOA, MAOB, and PLCB2, which may be commonly involved in the signaling of multiple neurotransmitter systems, rather than with canonical dopamine-specific proteins that directly bind dopamine (Figure 5). In contrast, aconitine and spinosin were predicted to potentially interact directly with calcium or sodium channels, indicating that their potential sites of action affecting the dopaminergic synapse were distinct from ephedrine (Figure A4).

## 3. Discussion

### 3.1. Summary of the Findings

This study identified five adverse event signals associated with EH-containing herbal medicine products through a disproportionality analysis of the KAERS database: sleep disorder, dry mouth, insomnia, palpitations, and constipation. Four signals were detected in the primary analysis (sleep disorder, dry mouth, insomnia, and palpitations), while the sensitivity analysis identified four signals (dry mouth, insomnia, palpitations, and constipation). NP analysis revealed that these adverse events are mediated through three key pathways—Neuroactive ligand-receptor interaction, Pathways of neurodegeneration, and Dopaminergic synapse—providing molecular-level insights into the mechanisms underlying the observed clinical safety signals.

### 3.2. Disproportionality Signals and Clinical Implications

Although gastrointestinal disorders were the most frequently reported adverse event category in absolute terms, the signal detection analysis identified neuropsychiatric and cardiovascular events as signals of disproportionate reporting. The identified adverse event signals align with the known pharmacological profile of ephedrine, the primary active constituent of EH, which acts as a sympathomimetic agent (Figure 2) [5,10]. The neuropsychiatric symptoms (insomnia and sleep disorder) and the cardiovascular symptom (palpitations) represent the expected reactions consistent with sympathetic nervous system stimulation reported in previous studies [5,10,19]. Dry mouth, while recognized as a minor symptom in clinical trials of ephedra-containing supplements [20], showed a notably strong signal in our analysis. Three signals (dry mouth, insomnia, and palpitations) remained consistent across both the primary and sensitivity analyses. Clinicians should be aware of potential neuropsychiatric and cardiovascular adverse events when prescribing EH-containing products, particularly in patients with preexisting sleep disorders or cardiovascular conditions.

### 3.3. Molecular Mechanisms of EH-Associated Adverse Events

Based on the ORA results using the co-occurring side effect-related genes of EH, three major pathways were predicted: Neuroactive ligand–receptor interaction [21], Pathways of neurodegeneration [22], and Dopaminergic synapse [23] (Figure 3A, Table A5). These findings suggest that the adverse events induced by EH may be attributed to CNS mechanisms. These findings are consistent with the known pharmacokinetic properties of ephedrine. Ephedrine is known to cross the blood–brain barrier and exert pharmacological effects within the central nervous system [24,25]. Its relatively low molecular weight and sufficient lipophilicity facilitate penetration into the central nervous system [26]. Following absorption and penetration across the blood–brain barrier, ephedrine can reach brain tissue and stimulate central adrenergic pathways, which may contribute to CNS-related adverse events such as insomnia and anxiety [24]. Therefore, the enrichment of CNS-related pathways identified in this study is consistent with the known pharmacological properties of ephedrine. Analysis of the PPI network showed that the Dopaminergic synapse exhibited the highest centrality values among the three pathways across all centrality metrics except degree centrality at the highest confidence threshold, suggesting its high potential to act as a hub within the adverse event-related system of EH (Table A6). In contrast, most proteins were unique to each of the three pathways, indicating that the pathways represent distinct molecular components within the adverse event-related PPI network (Figure A1). Taken together, the high centrality of the Dopaminergic synapse may be interpreted as reflecting its relatively central position within the overall network topology rather than resulting from extensive protein overlap with the other pathways. This suggests that the Dopaminergic synapse may be an important pathway for understanding the potential mechanisms underlying EH-associated adverse events. Recent studies have reported that the dopaminergic pathway regulates not only the CNS but also a wide range of physiological and pathological systems [27]. Consistently, the present study showed that the dopaminergic pathway may act as a central network role regulating neural responses within the side effect system (Table A6).

Investigation of the proportion of side effect-related proteins overlapping with each pathway showed that, except for sleep-related effects directly associated with dopamine, most adverse events were mainly enriched in the Neuroactive ligand–receptor interaction and Pathways of neurodegeneration, suggesting that these pathways may act as key sites for the mechanism underlying adverse events (Table A7, Figure 3B,C and Figure A2). Interestingly, of the three pathways, the Pathways of neurodegeneration showed an overwhelmingly high hit ratio of side effect-related proteins in dry mouth alone. This finding suggests a potential association between dry mouth and the involvement of neurodegeneration-related mechanisms in EH-associated adverse events [28]. However, because not all cases of dry mouth are associated with neurodegeneration, this finding should not be overgeneralized, and further studies are required. Nevertheless, because dry mouth is a symptom that can be relatively easily recognized by patients in daily life, its potential utility as an easily observable clinical signal warrants further investigation if its association with neurodegeneration-related changes is confirmed in future studies.

Exploratory docking analysis predicted that ephedrine, aconitine, and spinosin were likely to interact with distinct proteins (Figure 4 and Figure A3). In particular, ORA using proteins predicted to interact specifically with ephedrine showed that ephedrine had a relatively higher potential to act on the Dopaminergic synapse compared with the other two compounds (Figure A3B). In the preceding network analysis, we identified the Dopaminergic synapse as a potential key pathway in EH-associated adverse events, and the exploratory docking analysis showed a consistent trend.

We further examined the predicted direct molecular interactions of the three compounds within the Dopaminergic synapse pathway (Figure 5 and Figure A4). The three compounds tended to act through distinct mechanisms. Notably, GNAL, GNAS, PLCB2, MAOA, MAOB, PPP2R5A, and PPP2R5B, which were predicted to interact with ephedrine, are not receptors that directly bind dopamine, such as DRDs, or ion channels that directly regulate ion influx. A common feature of these proteins is that they can be involved in multiple neurotransmitter systems (Figure 5). GNAL and GNAS are heterotrimeric G-protein α subunits, whereas PLCB2 is a phospholipase C signaling effector. These proteins are not restricted to dopamine-specific signaling but can participate in GPCR signal transduction associated with multiple neurotransmitter receptors [29,30]. MAOA and MAOB are not enzymes that selectively metabolize dopamine but are involved in the metabolism of multiple neurotransmitters, including serotonin, dopamine, norepinephrine, and epinephrine [31]. In addition, PPP2R5A and PPP2R5B are PP2A regulatory subunits that regulate intracellular protein dephosphorylation and are involved in a broad range of biological processes [32]. Taken together, these findings suggest that ephedrine may contribute to CNS-related adverse events by broadly perturbing multiple neurotransmitter systems, including the dopaminergic system. However, the KEGG pathway library used in this study does not include separate neurotransmitter-specific pathways for other neurotransmitters, such as serotonin or epinephrine. Therefore, the composition of the pathway library may have influenced the identification of the Dopaminergic synapse as the only major neurotransmitter-specific pathway. In contrast, the other two compounds were predicted to directly interact with ion channels involved in ion regulation within the dopaminergic synapse (Figure A4). Specifically, aconitine was predicted to interact with calcium channels, whereas spinosin was predicted to interact with sodium channels, distinguishing them from ephedrine in that ion channels were identified as their potential sites of action. This exploratory docking-based approach may therefore be useful for complementing network-based analyses by identifying potential sites of compound action that are difficult to specifically determine from PPI network analysis. However, a limitation of these analyses is that they cannot clearly determine whether the corresponding neurotransmitter systems are hyperactivated or hypoactivated. Therefore, future studies should further develop this mechanism-based adverse event prediction model through experimental validation that can determine the direction of these effects.

### 3.4. Limitations and Future Directions

This study has several limitations. First, as with all disproportionality analyses, it cannot establish causality or measure incidence rates. Spontaneous reporting systems are subject to underreporting, selective reporting, and reporting bias [33]. In addition, reporting of EH-associated adverse events may have been influenced by notoriety bias related to the known safety concerns of ephedrine-containing products [34]. Second, the reference group consisted of 644 reports representing the entirety of available adverse event reports for herbal medicine products within our database extract. This reference group size reflects the limited number of herbal medicine formulas commercially regulated within this data source, rather than a sampling choice. The limited size of this reference group reduces the precision of disproportionality estimates, particularly for adverse events with low absolute counts [35]. In addition, this comparator group comprises a heterogeneous mixture of herbal medicine products with diverse compositions and indications, which may introduce confounding when interpreting differences in adverse event patterns relative to EH-containing products. Third, the external validity is limited by the demographic characteristics of our study population (predominantly adults aged 19–64 years, 60.4% female) and the specific traditional East Asian herbal medicine products analyzed. Additionally, EH-related reports had missing data for age (29.0%) and sex (27.4%), which is a limitation of spontaneous reporting systems and may affect the completeness of demographic characterization. Notably, sex was missing far more often in EH-related reports (27.4%) than in comparator reports (8.2%), suggesting this missingness is unlikely to be completely at random; the reported female-to-male ratio should therefore be interpreted with caution. Fourth, because EH-containing products are polyherbal formulations, the observed adverse events may reflect contributions from multiple herbal constituents rather than EH alone. In traditional East Asian herbal medicine practice, EH is rarely administered as a single herb and is instead used as a component of multi-herb formulas. A post-hoc exploratory analysis found no significant correlation between ingredient overlap with comparator formulas and signal rate (Dataset S6), providing no clear evidence that ingredient overlap alone explains the observed signals; however, this analysis does not rule out ingredient-level confounding. Fifth, the analysis was conducted using only the representative compounds of each herbal medicine. Therefore, it may not fully reflect the overall pharmacological mechanisms of the herbs. When multiple compounds are used together, unexpected adverse events may arise depending on the combination of target proteins with which the individual compounds interact. Indeed, previous studies have reported that the likelihood of adverse events may increase when multiple compounds act simultaneously on the same target proteins within disease-related protein networks [36]. Therefore, if identical compounds or structurally similar compounds present in a polyherbal formulation act on the same target protein [37], stimulation of that protein may exceed the activation threshold required under normal physiological conditions, which may increase the likelihood of adverse events. However, because the present study analyzed only representative compounds that were considered to be most closely associated with the adverse events, such potential multi-compound interactions were not considered. Specifically, this study focused on ephedrine, aconitine, and spinosin as representative compounds for the analysis. Ideally, all compounds present in each herbal medicine, as well as their potential interactions, should be considered. For example, pseudoephedrine is also recognized as a major alkaloid present in EH, and its contribution to the observed adverse-event mechanisms cannot be excluded because it may exert sympathomimetic effects similar to those of ephedrine [38]. However, the computational cost associated with large-scale multi-compound combinations remains substantial, and standardized methodologies for systematically evaluating interactions among multiple compounds have not yet been fully established. Therefore, the present study performed an exploratory analysis using ephedrine, which is representative of the adverse-effect profile of EH [39], together with comparator compounds exhibiting either similar or opposing pharmacological properties. Future studies should develop systematic approaches for evaluating compound–compound interactions while simultaneously considering a broader range of constituents that may contribute to adverse events. Sixth, because this study was conducted based on database-driven analyses, it may be affected by limitations of the currently available databases. For example, although “palpitations” was identified as a significant pharmacovigilance signal, no curated gene associations were available in the DisGeNET database version used in this study. Consequently, this clinically important adverse event could not be incorporated into the downstream network pharmacology analysis. In addition, because docking analysis in this study was performed using AutoDock Vina (v1.2.0) with the receptor treated as rigid, the analysis did not account for receptor flexibility. In particular, rigid docking using AlphaFold structures may not sufficiently capture changes in binding sites and ligand–protein interactions associated with receptor conformational changes in proteins such as GPCRs and ion channels. Finally, we were unable to perform additional experimental validation in the present study. Although data-driven analyses generate predictions based on existing biological knowledge and accumulated experimental evidence, caution should be exercised when interpreting the predicted results as direct molecular mechanisms in the absence of experimental validation. However, the primary objective of this study was to analyze EH-related adverse events and generate hypotheses regarding their potential molecular mechanisms. Nevertheless, experimental studies could provide stronger evidence supporting the proposed mechanisms. Therefore, future studies should experimentally validate the key proteins and signaling pathways predicted in the present study. Despite these limitations, this study is important because it explored the molecular mechanisms underlying the adverse events of EH, starting from clinical case reports of EH-associated adverse events. Notably, it provides novelty by integrating clinical data with cellular-level analyses of side effect mechanisms, which has rarely been attempted. Furthermore, this work establishes an analytical foundation that can be extended to predict adverse events and evaluate the safety of herbal combinations in future research. If future studies perform additional validation approaches, such as experimental validation and validation using known ligand–target complexes, more definitive conclusions may be achieved, which could contribute to the advancement of adverse event research.

## 4. Materials and Methods

### 4.1. Study Design

This research integrated three approaches to achieve the study aims: (1) disproportionality analysis of ICSRs using the KAERS database (2012–2021) to identify adverse event signals, (2) adverse event-associated NP analysis to explore biological pathways and construct PPI networks, and (3) molecular docking analysis to predict adverse event-associated molecular targets of ephedrine and compare the mechanisms with control compounds (aconitine and spinosin). This study is reported according to the Reporting of a Disproportionality Analysis for Drug Safety Signal Detection Using Individual Case Safety Reports in PharmacoVigilance (READUS-PV) statement [40].

### 4.2. Disproportionality Analysis Using the KAERS Database

#### 4.2.1. Data Source

The KAERS database, managed by the Korea Institute of Drug Safety and Risk Management (KIDS), is a nationwide pharmacovigilance system that collects postmarketing adverse event reports. This study utilized KAERS data for 84 herbal medicine products reported between 2012 and 2021 (KIDS KAERS DB (2205A0042)). These 84 herbal medicine formulas, encompassing 4894 approved products and selected based on national health insurance coverage, production volume, classification as basic formulas in standard textbooks, and clinical usage frequency [12], represent virtually the complete population of commercially manufactured herbal medicine products in Korea. The database contains ICSRs with information on patient demographics, drug details, adverse events coded using the Medical Dictionary for Regulatory Activities (MedDRA), and causality assessments.

#### 4.2.2. Data Extraction and Preprocessing

ICSRs for 84 herbal medicine products reported between 2012 and 2021 were extracted from the KAERS database. Of these products, 53 had one or more ICSRs reported. Duplicate reports were identified based solely on a case-grouping identifier in the KAERS database that links an original report to its follow-up reports for the same case. For each group of linked reports, only the report with the most recent follow-up sequence number was retained, and earlier versions were excluded as duplicates. Reports with missing adverse event names were also excluded. EH-containing herbal medicine products were identified through the Ministry of Food and Drug Safety’s public database (http://nedrug.mfds.go.kr, accessed on 13 July 2025). The seven identified EH-containing products are approved formulations with fixed compositions, as listed in Table A1.

#### 4.2.3. Study Population and Variables

The study population consisted of patients who reported adverse events associated with EH-containing herbal medicine products. Reports with EH as a suspected drug as well as those with EH as a concomitant medication were included in the primary analysis, reflecting the fact that the reporter-assigned suspected/concomitant designation does not necessarily correspond to the true pharmacological contribution of each component. To assess the robustness of the detected signals to this inclusion, a sensitivity analysis restricted to reports in which EH was the sole suspected drug was additionally conducted. Adverse events were coded using MedDRA version 26.0. Events were reported as MedDRA Lowest Level Terms and converted to PT. Key variables included reporter characteristics, patient demographics, and adverse event characteristics (SOC, PT, seriousness).

#### 4.2.4. Statistical Analysis

Descriptive statistics were used to characterize the baseline features of the reports, with categorical variables presented as frequencies and percentages. The most frequently reported adverse events were identified and ranked. Disproportionality analysis was conducted to detect signals of disproportionate reporting for EH-containing products compared with other herbal medicine products. A 2 × 2 contingency table was constructed with EH-containing products as the drug of interest and other herbal products as comparators. Three measures were calculated: PRRs, RORs, and ICs, all with 95% confidence intervals (CIs). Signal detection criteria were defined as a lower limit of the ROR 95% CI ≥ 1 and a lower limit of the IC 95% CI > 0 [41]. No multiple testing correction was applied, as this study was conducted as an exploratory, hypothesis-generating analysis intended to identify candidate safety signals for further investigation rather than to test a pre-specified hypothesis in a confirmatory framework. The sensitivity analysis (Section 4.2.3) excluded reports with concomitant medications. A standardized case-by-case causality assessment was not independently conducted by the authors due to the limited clinical details in the ICSRs; causality information, where available, reflects the assessment recorded by the original reporter in the KAERS database. Statistical analyses were performed using R version 4.4.3, and disproportionality analysis was conducted using the pvda package [42].

### 4.3. Construction and Analysis of Biological Protein–Protein Interaction (PPI) Networks Related to EH-Associated Adverse Events

#### 4.3.1. Collection of Biological Entities Related to Adverse Events

Biological entities reported to be related to adverse events that were statistically significant in spontaneous adverse event reports of EH were collected from the DisGeNET database [43] (accessed 10 May 2022). The DisGeNET database was accessed using a publicly available FTP release containing the complete dataset because recent versions of DisGeNET no longer publicly distribute the complete dataset via FTP. From the “all_gene_disease_associations” dataset provided by DisGeNET, genes associated with the EH-related adverse events (insomnia, palpitations, dry mouth, constipation, and sleep disorder) were retrieved by searching each adverse event as a disease term. Dry mouth, clinically referred to as xerostomia, was searched for using the disease term “Xerostomia.” For consistency and readability, the term “dry mouth” is used throughout this manuscript. To maintain consistency throughout the analysis and minimize potential selection bias by applying the same database and gene selection criteria, adverse events for which no associated genes were identified in the DisGeNET database were excluded from the analyses. To enhance the sensitivity and interpretability of the analysis, all disease terms containing the same keyword (e.g., cluster headache and chronic headache) were grouped into a single side effect group. In this process, duplicate genes within each side effect group were normalized as unique entries for subsequent analyses.

Genes included in each side effect group were used to analyze the biological pathways associated with adverse events. To identify co-occurring adverse events of EH, genes that were included in two or more side effect groups were defined as co-occurring side effect-related genes. These genes were analyzed using ORA [44], which was performed using the Enrichr platform [45] (https://maayanlab.cloud/Enrichr/, accessed 4 September 2025) with the gene sets provided in the KEGG_2021_Human library [46]. Among the significantly enriched pathways, three pathways directly related to EH were selected for subsequent analyses based on an adjusted *p*-value < 0.001. These pathways were included in the subsequent analyses because they reflect CNS functions that are closely associated with the known pharmacological actions of EH and its adverse events.

#### 4.3.2. Construction and Analysis of an Adverse Events-Related Biological PPI Network

KEGG pathway-based physiological PPI network related to the adverse events of EH was constructed using the EH side effect-related pathways. This biological PPI network was built using the KEGG_2021_Human library provided by the EnrichR platform and the PPI scores provided by the STRING database [47] (v12, accessed 5 March 2024). The nodes of the PPI network were defined as genes belonging to the three pathways in the KEGG_2021_Human library. Edges were defined between node pairs with an interaction score greater than 900, corresponding to the highest confidence criterion provided by the STRING database. Nodes without edges were excluded from the network. To examine the network structure and distribution of the gene sets, color mapping was applied to the PPI network. To identify which pathways played a hub role within the network, the average values of degree centrality, betweenness centrality, and closeness centrality were calculated for the genes belonging to each pathway [48]. Because centrality metrics were calculated within a single integrated PPI network representing EH-related adverse events, rather than within individual pathway subnetworks, no pathway size or edge-density normalization and statistical comparison were performed. Subsequently, the number of overlapping genes between pathways was counted to examine how different pathways interacted with each other during the occurrence of adverse events. Furthermore, the overlap between side effect-related gene sets and each pathway was quantified by calculating the number of side effect-related genes included in each pathway relative to the total number of genes associated with each side effect. This analysis was performed to identify which adverse events were most closely associated with each pathway. Additional sensitivity analyses were performed to evaluate whether the network constructed in this study was dependent on a specific interaction score threshold. The high confidence (700) and medium confidence (400) thresholds provided by STRING were applied, and the networks were constructed and analyzed using the same procedures as those used for the 900 threshold to evaluate the robustness of the network. The network visualization was performed by color-mapping the adverse event-related genes in the PPI network constructed at the 900 threshold, while the networks constructed at the 400 and 700 thresholds were provided as a Supplementary Dataset (Supplementary Datasets S3 and S4). Data preprocessing and analyses were performed using Python (v3.10.12) and the NetworkX library [49] (v3.2.1), while centrality analysis and visualization were conducted using CytoScape software [50] (v3.10.1).

### 4.4. Exploratory Docking Screening and Identification of Targets Using the Major Compound of EH

#### 4.4.1. Exploratory Docking Screening Method Using the Major Compound of EH and Control Compounds

An exploratory docking screening was performed using ephedrine, the representative compound of EH, to predict its potential for direct molecular interactions. As control compounds, aconitine, a constituent of AL known to stimulate the sympathetic nervous system, and spinosin, a constituent of ZS known to suppress the sympathetic nervous system, were used. Aconitine and spinosin were selected as comparator compounds because they are representative herbal-derived compounds with clearly distinct safety and pharmacological profiles. Aconitine is a well-known toxic alkaloid associated with cardiovascular and neurological adverse effects, similar to ephedrine [51]. In contrast, spinosin, a major bioactive constituent of ZS, is well known for its calming and sleep-promoting effects and exhibits pharmacological properties opposite to those of ephedrine [52]. Therefore, these compounds were included as reference compounds for the comparative interpretation of the molecular mechanisms underlying ephedrine-related adverse events. Specifically, aconitine was included to determine whether ephedrine shares common molecular mechanisms with another toxic alkaloid or possesses distinct ephedrine-specific mechanisms. In addition, spinosin was selected to evaluate whether pharmacological effects opposite to those of ephedrine are associated with opposing molecular mechanisms or arise through independent mechanisms, using exploratory docking analysis.

The compounds used for docking analysis were obtained from the PubChem database [53] as 3D SDF files (PubChem CIDs: 9294 (ephedrine), 245005 (aconitine), and 155692 (spinosin), accessed 4 September 2025). Each compound was converted from SDF to PDBQT format using OpenBabel GUI software [54] (v3.1.1) for docking preparation. The default settings of OpenBabel GUI software were used during ligand file conversion. Accordingly, no additional water molecules were added, and no protonation-state optimization was performed.

The protein set used for the exploratory docking screening was derived from the 762 nodes included in the side effect-related biological PPI network. Protein structures were obtained from the AlphaFold database [55] (AlphaFold v2, https://ftp.ebi.ac.uk/pub/databases/alphafold/v2/, accessed on 4 September 2025), using human protein information from the reference proteome (ID: UP000005640). We used the AlphaFold database to obtain full-length protein structures in our analysis. In addition, AlphaFold structures are consistently centered, making them suitable for applying uniform docking parameters in large-scale exploratory docking analyses. Both the 3D PDB structure files of the proteins and fractions included in the network nodes were downloaded. The downloaded protein structure files were converted to PDBQT format using the Python OpenBabel library (v3.1.0), and torsion information was removed to generate rigid forms. Because the protein structures provided by AlphaFold do not contain explicit water molecules, no additional water-molecule processing was performed.

Large-scale exploratory docking screening was performed using the Python AutoDockVina library [56] (v1.2.0). Docking analyses were conducted for all possible pairs between the three compounds converted to PDBQT format and the protein set. The docking parameters were set as follows: center = (0, 0, 0), box size = 126, exhaustiveness = 100, and n_poses = 5. Of the five poses, the lowest binding affinity score was selected as the docking result.

#### 4.4.2. Docking and Network Analyses of Adverse Events-Related Targets

The results of the docking analysis were used to predict the major targets with the highest potential for direct molecular interactions. To account for differences in molecular size and the distribution of binding affinities among compounds, docking scores were normalized using the SILE method [57]. Because larger molecules generally exhibit higher binding affinities, the concept of ligand efficiency, which evaluates binding energy on a per-atom basis, has been proposed to correct for molecular size bias [58]. Nissink et al. reported that SILE (affinity/N^0.3^, where N is the number of heavy atoms excluding hydrogen atoms) more effectively corrects the molecular size bias observed in conventional ligand efficiency metrics [57]. Accordingly, affinity scores were normalized using the SILE equation in the present study. Empirical *p*-values were subsequently calculated from the normalized scores, and proteins with an empirical *p*-value < 0.05 were selected as candidate major interaction targets. For each of the three compounds, the top 5% of proteins with the highest interaction potential, corresponding to an empirical *p*-value of 0.05, were selected as candidate major interaction targets. Because the candidate target proteins function within the biological side effect-related PPI network through direct protein interaction cascades or indirect information flows, only the 762 proteins included in the PPI network were considered. In the AlphaFold database, some proteins are represented by a single fraction, whereas others are divided into multiple fractions. Therefore, after applying the empirical *p*-value threshold, when multiple protein fractions corresponded to the same protein, only the fraction with the highest interaction potential was retained. To determine whether the proteins predicted to interact with each compound exhibited hub characteristics within the PPI network, the average values of degree centrality, betweenness centrality, and closeness centrality were calculated for the genes belonging to each pathway. As in the previous analysis, additional sensitivity analyses were performed using the high confidence (700) and medium confidence (400) thresholds to evaluate the robustness of the results and to assess whether the hub characteristics of each compound were consistently observed across different confidence thresholds. As in the pathway analysis, the number of overlapping genes between pathways was counted to evaluate whether each compound acted independently. In addition, the distribution ratio of major interacting proteins across pathways was calculated to identify which pathways were related to each compound.

The major target proteins were visualized by color mapping in the network. From the matrix containing the major interaction results represented as one-hot encoded values, Boolean masking was applied to filter only the major interacting proteins of each compound, and these matrices were used for visualization. Finally, the predicted sites of action of the three compounds were compared within the dopaminergic synapse pathway, which was predicted to be specifically associated with ephedrine. Proteins predicted as unique major targets for each compound were highlighted in red to visualize their putative sites of action within the pathway. In addition, canonical dopamine-specific proteins were highlighted in blue to distinguish dopamine-specific signaling from general neurotransmitter signaling, allowing us to evaluate whether the putative actions of ephedrine were dopamine-specific or involved broader neurotransmitter-related mechanisms. Centrality analysis and network visualization were performed using CytoScape software, matrix visualization was performed using the Python Seaborn library [59] (v0.13.2), and dopaminergic synapse pathway visualization was performed using the KEGG Mapper platform [60].

## 5. Conclusions

This study identified and characterized EH-associated adverse event signals by integrating clinical pharmacovigilance data from the KAERS database. Additionally, we applied data-driven analyses based on existing biological knowledge and accumulated experimental evidence to predict the molecular mechanisms most likely to underlie EH-associated adverse events. Disproportionality analysis revealed significant signals, including insomnia, dry mouth, and palpitations, which are consistent with sympathetic nervous system stimulation. Network analysis further predicted that these adverse events are most likely to be mediated through three key pathways: Neuroactive ligand-receptor interaction, Pathways of neurodegeneration, and Dopaminergic synapse. Furthermore, molecular docking predicted that ephedrine is the compound most likely to modulate the dopaminergic synapse through a mechanism distinct from those of the comparator compounds. Moreover, its putative actions were predicted not to be restricted to dopamine-specific mechanisms but to extend to general neurotransmitter signaling.

## Figures and Tables

**Table 1:** Baseline characteristics of reports of EH-containing HMPs.

Report Information	ICSRs of Ephedra-Containing HMPs (*n* = 379)	ICSR of Other HMPs (*n* = 644)	Total (*n* = 1023)
Original reporter			
Doctor, dentist, Korean medicine doctor	39 (10.3)	117 (18.2)	156 (15.2)
Pharmacist, Korean medicine pharmacist	230 (60.7)	238 (37.0)	468 (45.7)
Other medical professional	11 (2.9)	54 (8.3)	65 (6.4)
Consumer, nonmedical professional	72 (19.0)	221 (34.2)	293 (28.6)
Unknown	27 (7.1)	14 (2.2)	41 (4.0)
Reporter			
Pharmaceutical company	133 (35.1)	218 (33.9)	351 (34.3)
Medical expert (e.g., hospital, pharmacy)	30 (7.9)	71 (11.0)	101 (9.9)
Regional pharmacovigilance center	193 (50.9)	273 (42.4)	466 (45.6)
Other (e.g., distributor)	4 (1.1)	38 (5.9)	42 (4.1)
Patient, consumer	19 (5.0)	44 (6.8)	63 (6.2)
Report type			
Voluntary reporting	352 (92.9)	590 (91.6)	942 (92.1)
Reporting in trial/research	22 (5.8)	33 (5.1)	55 (5.4)
Other	5 (1.3)	19 (3.0)	24 (2.3)
Unknown	0 (0.0)	2 (0.3)	2 (0.2)
Patient demographics			
Age at the time of occurrence			
0–27 days	2 (0.5)	0 (0.0)	2 (0.2)
28 days to 1 year	2 (0.5)	2 (0.3)	4 (0.4)
2–11 years	2 (0.5)	4 (0.6)	6 (0.6)
12–18 years	3 (0.8)	7 (1.1)	10 (1.0)
19–64 years	232 (61.2)	342 (53.1)	574 (56.1)
65 years or older	28 (7.4)	124 (19.3)	152 (14.9)
(Missing)	110 (29.0)	165 (25.6)	275 (26.9)
Sex			
Male	46 (12.1)	218 (33.9)	264 (25.8)
Female	229 (60.4)	373 (57.9)	602 (58.8)
(Missing)	104 (27.4)	53 (8.2)	157 (15.3)

EH, Ephedrae Herba; ICSR, individual case safety report; HMPs, herbal medicine products.

**Table 2:** Characteristics of 559 adverse events with EH-containing herbal medicine products.

Adverse Events Classified by System Organ Class	*n* (%)
Gastrointestinal disorders	180 (32.2)
Nervous system disorders	75 (13.4)
Skin and subcutaneous tissue disorders	71 (12.7)
Psychiatric disorders	67 (12.0)
General disorders and administration site conditions	40 (7.2)
Investigations	33 (5.9)
Cardiac disorders	29 (5.2)
Metabolism and nutrition disorders	10 (1.8)
Eye disorders	8 (1.4)
Respiratory, thoracic, and mediastinal disorders	8 (1.4)
Musculoskeletal and connective tissue disorders	8 (1.4)
Injury, poisoning, and procedural complications	6 (1.1)
Renal and urinary disorders	6 (1.1)
Infections and infestations	5 (0.9)
Reproductive system and breast disorders	3 (0.5)
Ear and labyrinth disorders	2 (0.4)
Hepatobiliary disorders	2 (0.4)
Vascular disorders	2 (0.4)
Blood and lymphatic system disorders	2 (0.4)
Immune system disorders	1 (0.2)
Product issues	1 (0.2)
Serious adverse event	
SAE	16 (2.9)
Non-SAE	543 (97.1)

EH, Ephedrae Herba; SAE, serious adverse event.

**Table 3:** The 16 most frequently reported adverse events with EH-containing herbal medicine products.

System Organ Class	Adverse Event (PT)	*n* (%)
Psychiatric disorders	Insomnia	47 (8.4)
Gastrointestinal disorders	Abdominal discomfort	36 (6.4)
Nervous system disorders	Headache	28 (5)
Cardiac disorders	Palpitations	25 (4.5)
Gastrointestinal disorders	Diarrhea	24 (4.3)
Gastrointestinal disorders	Dry mouth	20 (3.6)
Skin and subcutaneous tissue disorders	Urticaria	19 (3.4)
Gastrointestinal disorders	Abdominal pain	18 (3.2)
Gastrointestinal disorders	Constipation	17 (3)
Gastrointestinal disorders	Dyspepsia	17 (3)
Gastrointestinal disorders	Nausea	17 (3)
Skin and subcutaneous tissue disorders	Pruritus	17 (3)
Nervous system disorders	Dizziness	16 (2.9)
Gastrointestinal disorders	Vomiting	13 (2.4)
Psychiatric disorders	Sleep disorder	11 (2)
Skin and subcutaneous tissue disorders	Rash	11 (2)

EH, Ephedrae Herba; PT, preferred term.

## Data Availability

The data analyzed in this study were obtained from the KIDS KAERS DB (2205A0042) and are not publicly available owing to the KIDS data protection policy. Requests to access the datasets should be directed to the KAERS (https://open.drugsafe.or.kr/, accessed on 29 July 2022). All datasets used for the in silico analyses are available in the Supplementary Materials.

## Supplementary Materials

The following supporting information can be downloaded at: https://www.mdpi.com/article/10.3390/ph19091340/s1, Dataset S1: Side effect-related gene symbol list; Dataset S2: Co-occurring side effect gene counts; Dataset S3: Network interaction information; Dataset S4: Network node information; Dataset S5: Docking result. Dataset S6: Ingredient overlap and signal rate data for comparator formulas.

## Abbreviations

The following abbreviations are used in this manuscript:

AL	Aconiti Lateralis Radix Preparata
ANS	autonomic nervous system
CI	confidence interval
CNS	central nervous system
EH	Ephedrae Herba
GPCR	G protein–coupled receptor
IC	information component
ICSRs	individual case safety reports
ITPR	inositol 1,4,5-trisphosphate receptor
KAERS	Korea Adverse Event Reporting System
KEGG	Kyoto Encyclopedia of Genes and Genomes
LLT	lowest level terms
MedDRA	Medical Dictionary for Regulatory Activities
NP	network pharmacology
ORA	Over-representation analysis
PLCB	phospholipase C beta
PPI	protein–protein interaction
PRR	proportional reporting ratio
PT	preferred terms
ROR	reporting odds ratio
SILE	Score-Integrated Ligand Efficiency
SOC	System Organ Class
ZS	Ziziphi Semen

## Appendix A

**Table A1:** List of seven EH-containing herbal medicine products with reported adverse events in KAERS database (2012–2021).

Product Name (Chinese, Japanese)	Herbal Composition	Number of ICSRs (%)
Bangpung-tongseong-san (Fangfengtongsheng-san, Bofutsusho-san)	Talcum 1.67 g, Glycyrrhizae Radix 0.67 g, Gypsum Fibrosum 1 g, Scutellariae Radix 0.67 g, Platycodonis Radix 0.67 g, Saposhnikoviae Radix 0.4 g, Paeoniae Radix 0.4 g, Cnidii Rhizoma 0.4 g, Angelicae Gigantis Radix 0.4 g, Rhei Radix et Rhizoma 0.5 g, Ephedrae Herba 0.4 g, Menthae Herba 0.4 g, Forsythiae Fructus 0.4 g, Natrii Sulfas 0.5 g, Schizonepetae Spica 0.4 g, Atractylodis Rhizoma Alba 0.67 g, Gardeniae Fructus 0.4 g, Zingiberis Rhizoma Recens 0.4 g	282 (74.4%)
Galgeun-tang (Gegen-tang, Kakkon-to)	Puerariae Radix 2.67 g, Ephedrae Herba 1.33 g, Cinnamomi Ramulus 1 g, Paeoniae Radix 1 g, Glycyrrhizae Radix 0.67 g, Zingiberis Rhizoma 0.33 g, Jujubae Fructus 1.33 g	49 (12.9%)
Socheongryong-tang (Xiaoqinglong-tang, Shoseiryu-to)	Ephedrae Herba 1 g, Paeoniae Radix 1 g, Asiasari Radix et Rhizoma 1 g, Zingiberis Rhizoma 1 g, Glycyrrhizae Radix 1 g, Cinnamomi Ramulus 1 g, Pinelliae Tuber 2 g, Schisandrae Fructus 1 g	24 (6.3%)
Ojeok-san (Wuji-san, Goshaku-san)	Atractylodis Rhizoma 1.33 g, Ephedrae Herba 0.67 g, Citri Unshius Pericarpium 0.67 g, Magnoliae Cortex 0.67 g, Platycodonis Radix 0.67 g, Aurantii Fructus Immaturus 0.67 g, Angelicae Gigantis Radix 0.67 g, Zingiberis Rhizoma 0.67 g, Paeoniae Radix 0.67 g, Poria Sclerotium 0.67 g, Angelicae Dahuricae Radix 0.67 g, Cnidii Rhizoma 0.67 g, Pinelliae Tuber 0.67 g, Cinnamomi Ramulus 0.67 g, Glycyrrhizae Radix 0.67 g, Jujubae Fructus 0.67 g, Cyperi Rhizoma 0.4 g	16 (4.2%)
Yeongsen-jetong-eum (-, -)	Ephedrae Herba 1.25 g, Paeoniae Radix 1.25 g, Saposhnikoviae Radix 0.625 g, Schizonepetae Spica 0.625 g, Osterici Radix 0.625 g, Araliae Continentalis Radix 0.625 g, Clematidis Radix 0.625 g, Angelicae Dahuricae Radix 0.625 g, Atractylodis Rhizoma 0.625 g, Scutellariae Radix 0.625 g, Aurantii Fructus Immaturus 0.625 g, Platycodonis Radix 0.625 g, Puerariae Radix 0.625 g, Cnidii Rhizoma 0.625 g, Angelicae Gigantis Radix 0.375 g, Cimicifugae Rhizoma 0.375 g, Glycyrrhizae Radix 0.375 g	5 (1.3%)
Oyaksungi-san (Wuyaoshunqi-san, Uyakujyunki-san)	Ephedrae Herba 1.88 g, Citri Unshius Pericarpium 1.88 g, Linderae Radix 1.88 g, Cnidii Rhizoma 1.25 g, Angelicae Dahuricae Radix 1.25 g, Bombycis Corpus 1.25 g, Aurantii Fructus Immaturus 1.25 g, Platycodonis Radix 1.25 g, Zingiberis Rhizoma 0.63 g, Zingiberis Rhizoma Recens 0.50 g, Jujubae Fructus 0.67 g, Glycyrrhizae Radix 0.38 g	2 (0.5%)
Mahwang-tang (Mahuang-tang, Mao-to)	Ephedrae Herba 1.67 g, Cinnamomi Ramulus 1.33 g, Armeniacae Semen 1.67 g, Glycyrrhizae Radix 0.67 g	1 (0.3%)

EH, Ephedrae Herba; ICSRs: Individual Case Safety Reports.

**Table A2:** Characteristics of the 16 serious adverse events associated with EH-containing herbal medicine products.

SOC	PT	Seriousness Criteria	Outcome	Causality
Eye disorders	Angle closure glaucoma	Other important medical event	Recovered	Not assessed
Eye disorders	Vision blurred	Other important medical event	Recovered	Not assessed
Gastrointestinal disorders	Dry mouth	Other important medical event	Unknown	Not assessed
Gastrointestinal disorders	Nausea	Hospitalization	Not recovered	Not assessed
Gastrointestinal disorders	Nausea	Life-threatening, Hospitalization	Recovering	Possible
Gastrointestinal disorders	Retching	Other important medical event	Unknown	Not assessed
General disorders and administration site conditions	Asthenia	Hospitalization	Not recovered	Not assessed
Hepatobiliary disorders	Hepatitis acute	Hospitalization	Not recovered	Not assessed
Hepatobiliary disorders	Jaundice	Hospitalization	Not recovered	Not assessed
Investigations	Liver function test abnormal	Hospitalization	Recovered	Unable to assess
Metabolism and nutrition disorders	Cachexia	Hospitalization	Not recovered	Possible
Nervous system disorders	Dizziness	Other important medical event	Unknown	Not assessed
Nervous system disorders	Headache	Other important medical event	Recovered	Not assessed
Psychiatric disorders	Hallucination	Other important medical event	Unknown	Not assessed
Psychiatric disorders	Hallucination, auditory	Other important medical event	Unknown	Not assessed
Respiratory, thoracic and mediastinal disorders	Dyspnoea	Life-threatening, Hospitalization	Recovered	Probable

EH, Ephedrae Herba; SOC, System Organ Class; PT, preferred terms.

**Table A3:** Signal detection results for the 16 most frequently reported adverse events associated with EH-containing herbal medicine products.

Adverse Event (PT Level)	n	PRR	ROR (95% CI)	IC (95% CI)
Sleep disorder	11	9.35	9.60 (2.12, 43.52)	1.11 (0.14, 1.84)
Dry mouth	20	6.8	7.12 (2.65, 19.13)	1.07 (0.37, 1.63)
Insomnia	47	6.14	6.87 (3.67, 12.88)	1.06 (0.62, 1.45)
Palpitations	25	3.86	4.06 (1.98, 8.36)	0.88 (0.26, 1.39)
Constipation	17	2.89	2.98 (1.35, 6.57)	0.74 (−0.03, 1.34)
Headache	28	2.16	2.26 (1.27, 4.00)	0.58 (−0.00, 1.07)
Abdominal discomfort	36	1.85	1.94 (1.19, 3.17)	0.49 (−0.02, 0.92)
Abdominal pain	18	1.27	1.29 (0.69, 2.41)	0.20 (−0.54, 0.79)
Nausea	17	0.9	0.90 (0.49, 1.64)	−0.09 (−0.86, 0.51)
Dyspepsia	17	0.88	0.87 (0.48, 1.58)	−0.12 (−0.89, 0.48)
Vomiting	13	0.74	0.73 (0.37, 1.41)	−0.28 (−1.17, 0.39)
Dizziness	16	0.73	0.72 (0.40, 1.32)	−0.29 (−1.08, 0.33)
Diarrhoea	24	0.59	0.56 (0.35, 0.91)	−0.51 (−1.15, 0.01)
Pruritus	17	0.48	0.46 (0.26, 0.80)	−0.73 (−1.50, −0.13)
Urticaria	19	0.47	0.45 (0.26, 0.76)	−0.75 (−1.47, −0.17)
Rash	11	0.31	0.29 (0.15, 0.55)	−1.24 (−2.22, −0.51)

EH, Ephedrae Herba; PT, preferred terms; PRR, proportional reporting ratio; ROR, reporting odds ratio; IC, information component; CI, confidence interval.

**Table A4:** Sensitivity analysis: Signal detection results for the 16 most frequently reported adverse events associated with EH-containing herbal medicine products (suspected drug reports only).

Adverse Event (PT Level)	n	PRR	ROR (95% CI)	IC (95% CI)
Sleep disorder	8	13.18	13.48 (1.68, 108.26)	1.12 (−0.04, 1.95)
Dry mouth	14	11.53	11.99 (2.71, 53.11)	1.15 (0.29, 1.80)
Insomnia	40	7.32	8.18 (3.92, 17.09)	1.09 (0.61, 1.50)
Palpitations	25	4.58	4.87 (2.24, 10.56)	0.93 (0.31, 1.44)
Constipation	16	3.77	3.91 (1.59, 9.59)	0.84 (0.05, 1.47)
Abdominal discomfort	36	1.85	1.96 (1.19, 3.22)	0.48 (−0.03, 0.91)
Headache	24	1.8	1.86 (1.03, 3.37)	0.45 (−0.18, 0.97)
Abdominal pain	18	1.29	1.31 (0.69, 2.46)	0.21 (−0.53, 0.80)
Nausea	17	0.97	0.96 (0.52, 1.78)	−0.03 (−0.80, 0.57)
Dyspepsia	17	0.93	0.93 (0.50, 1.71)	−0.06 (−0.83, 0.54)
Vomiting	13	0.82	0.82 (0.41, 1.61)	−0.17 (−1.06, 0.50)
Diarrhoea	21	0.53	0.50 (0.30, 0.84)	−0.62 (−1.30, −0.07)
Dizziness	10	0.53	0.52 (0.25, 1.07)	−0.61 (−1.64, 0.15)
Pruritus	17	0.52	0.49 (0.28, 0.87)	−0.64 (−1.41, −0.04)
Urticaria	19	0.5	0.47 (0.27, 0.79)	−0.69 (−1.41, −0.11)
Rash	11	0.33	0.31 (0.16, 0.59)	−1.14 (−2.12, −0.42)

EH, Ephedrae Herba; PT, preferred terms; PRR, proportional reporting ratio; ROR, reporting odds ratio; IC, information component; CI, confidence interval.

**Table A5:** Top 10 enriched pathways identified by over-representation analysis using co-occurring adverse events-related genes.

Pathway Name	*p*-Value	Adjusted *p*-Value
Neuroactive ligand-receptor interaction	1.22 × 10^−13^	2.79 × 10^−11^
Pathways of neurodegeneration	3.03 × 10^−9^	3.46 × 10^−7^
Dopaminergic synapse	4.64 × 10^−9^	3.55 × 10^−7^
Alzheimer disease	0.00002289	0.00131
Human T-cell leukemia virus 1 infection	0.00004194	0.001921
Rheumatoid arthritis	0.00007174	0.002432
Circadian rhythm	0.00008169	0.002432
Calcium signaling pathway	0.00008497	0.002432
Spinocerebellar ataxia	0.0001035	0.002634
Inflammatory bowel disease	0.0001276	0.002922

**Table A6:** Results of centrality analysis of the three adverse event-related pathways in the protein–protein interaction network.

Confidence	Pathway Name	No. of Nodes	Degree Centrality	Betweenness Centrality	Closeness Centrality
Highest confidence (>900)	Neuroactive ligand-receptor interaction	288	7.1076	0.002	0.2432
	Pathways of neurodegeneration	410	22.1976	0.0048	0.2569
	Dopaminergic synapse	131	20.5954	0.0079	0.2912
High confidence (>700)	Neuroactive ligand-receptor interaction	324	14.2808	0.0015	0.3143
	Pathways of neurodegeneration	445	30.0225	0.003	0.3249
	Dopaminergic synapse	131	38.0305	0.0053	0.3659
Medium confidence (>400)	Neuroactive ligand-receptor interaction	338	51.3018	0.0011	0.4257
	Pathways of neurodegeneration	445	60.9011	0.0016	0.4371
	Dopaminergic synapse	131	94.4427	0.0035	0.4805

**Table A7:** Distribution of side effect-related proteins across the three adverse event-related pathways in the protein–protein interaction network.

Confidence	Pathway Name	Constipation	Insomnia	Sleep Disorder	Dry Mouth
Highest confidence (>900)	Neuroactive ligand-receptor interaction	37/76	13/35	35/64	1/15
	Pathways of neurodegeneration	35/76	17/35	21/64	15/15
	Dopaminergic synapse	12/76	10/35	13/64	0/15
High confidence (>700)	Neuroactive ligand-receptor interaction	37/76	13/35	36/66	1/16
	Pathways of neurodegeneration	35/76	17/35	22/66	16/16
	Dopaminergic synapse	12/76	10/35	13/66	0/16
Medium confidence (>400)	Neuroactive ligand-receptor interaction	37/76	13/35	39/69	1/16
	Pathways of neurodegeneration	35/76	17/35	24/69	16/16
	Dopaminergic synapse	12/76	10/35	13/69	0/16

**Table A8:** Results of centrality analysis of the direct interaction proteins predicted by docking analysis within the protein–protein interaction network.

Confidence	Compound Name	Degree Centrality	Betweenness Centrality	Closeness Centrality
Highest confidence (>900)	Ephedrine	14.4865	0.0062	0.2731
	Aconitine	15.0000	0.0083	0.2747
	Spinosin	16.8205	0.0051	0.2672
	Non-interaction nodes	16.5427	0.0037	0.2522
High confidence (>700)	Ephedrine	26.0541	0.0032	0.3459
	Aconitine	29.8974	0.0055	0.3511
	Spinosin	30.5128	0.0036	0.3416
	Non-interaction nodes	24.3699	0.0024	0.3213
Medium confidence (>400)	Ephedrine	70.0270	0.0019	0.4592
	Aconitine	82.1282	0.0031	0.4642
	Spinosin	66.5385	0.0016	0.4506
	Non-interaction nodes	58.6157	0.0015	0.4332
